# Novel temporary endodontic medication based on calcium silicate strategy: a biological and physicochemical study

**DOI:** 10.3389/fdmed.2024.1451275

**Published:** 2024-09-05

**Authors:** Claire El Hachem, Salvatore Sauro, Ammar Eid, Burçin Arıcan, Raya Alrayesse, Camille Fabro, Varvara Gribova, Louis Hardan, Youssef Haikel, Naji Kharouf

**Affiliations:** ^1^ Faculty of Dentistry, Saint Joseph University, Beirut, Lebanon; ^2^ Dental Biomaterials and Minimally Invasive Dentistry, Departamento de Odontología, Facultad de Ciencias de la Salud, Universidad CEU-Cardenal Herrera, Valencia, Spain; ^3^ Department of Therapeutic Dentistry, I. M. Sechenov First Moscow State Medical University, Moscow, Russia; ^4^ Department of Endodontics and Conservative Dentistry, Faculty of Dental Medicine, IUST University, Ghabagheb, Syria; ^5^ Department of Endodontics, Faculty of Dentistry, Bahçeşehir University, Istanbul, Türkiye; ^6^ Department of Endodontics and Conservative Dentistry, Faculty of Dentistry, Damascus University, Damascus, Syria; ^7^ Department of Biomaterials and Bioengineering, INSERM UMR_S 1121, Strasbourg University, Strasbourg, France; ^8^ Department of Endodontics and Conservative Dentistry, Faculty of Dental Medicine, Strasbourg University, Strasbourg, France; ^9^ Pôle de Médecine et Chirurgie Bucco-Dentaire, Hôpital Civil, Hôpitaux Universitaire de Strasbourg, Strasbourg, France

**Keywords:** calcium hydroxide cement, bioceramics, physicochemical properties, biological activity, crystallography

## Abstract

**Introduction:**

The purpose of this *in vitro* study was to compare the physicochemical and biological properties of a traditional calcium hydroxide-based cement (Cal) to a novel endodontic material based on calcium silicate strategy in premixed formulation (Bio-C).

**Methods:**

Crystalline structure evaluation and pH analysis were performed at different time periods (3–168 h). Contact angle, surface roughness, solubility and flowability of both materials were also investigated. The antibacterial activity of each material was assessed using a direct contact test against *Enterococcus faecalis (E.faecalis)*, and the cytotoxicity was performed by using periodontal ligament cells. Statistical analysis was accomplished using one-way analysis of variance and Kruskal-Wallis tests.

**Results:**

An alkaline pH was observed in both the tested materials. Higher pH values were attained in Cal compared to Bio-C (*p* < 0.05). Higher flowability, solubility and wettability were attained for Bio-C compared to Cal (*p* < 0.05). Crystalline structures were observed on the surface of Bio-C after immersion in PBS (168 h). Cal presented higher antibacterial activity compared to Bio-C against *E.faecalis*. Only undiluted Bio-C extracts demonstrated slight cytotoxicity, while all the other tested specimens had no cytotoxicity.

**Discussion:**

In conclusion, the novel bioceramic medication might be used as a suitable alternative agent to the traditional calcium hydroxide cements due to its biological and physicochemical properties. However, further studies on the ability of removing Bio-C from root canal are required to determine whether such a temporary bioceramic can ameliorate root canal treatments.

## Graphical Abstract

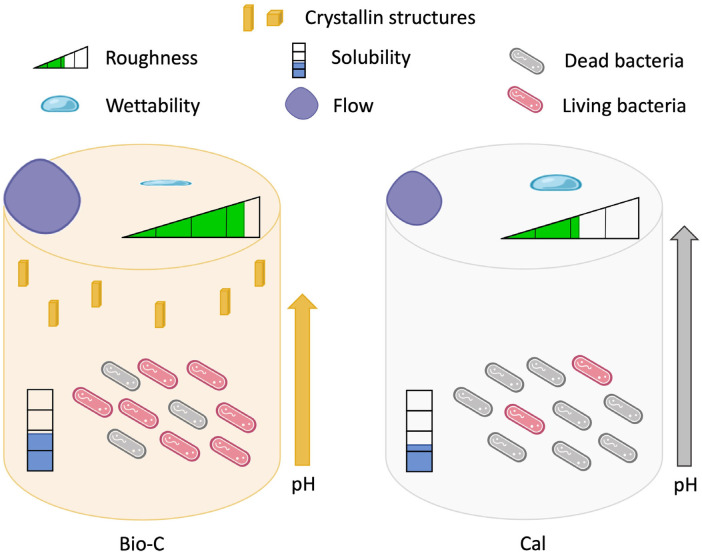

## 1 Introduction

In case of a severe infection of the root canal system, it may be necessary to treat the tooth in such a clinical scenario, it is recommended to place temporary medications as intracanal dressing between endodontics procedures performed at different appointments. The most commonly temporary material used today is calcium hydroxide [Ca(OH)_2_] (1).

The use of calcium hydroxide in endodontics began in 1920, when Hermann introduced it for the first time as a root canal filling material (2). Throughout the second part of the 20th century, it became widely popular because of its several benefits in endodontics. Even today, it is still extensively used and studied by many researchers (2). Calcium hydroxide is insoluble in alcohol, but it presents a low solubility in water (around 1.2 g L^−1^ at 25°C), which decreases when the temperature rises (1). The pure powder has the particularity of having a high pH, varying between 12.5 and 12.8 (3), which confers to it reliable antibacterial properties. Calcium hydroxide dissociates into calcium (Ca^2+^) and hydroxide (OH^−^) ions (1), which are slowly released when the Ca(OH)_2_ is in an aqueous medium and/or in contact with biological tissues (e.g., pulp tissue). The biological effects of calcium hydroxide can last for a prolonged period of time (4). In addition, due to its high pH level, the calcium hydroxide promotes active repair and calcification by preventing the decomposition of the mineral components of dentin. In some specific circumstances, It may induce the formation of complex calcium phosphates (e.g., hydroxyapatite) in the organic matrix and thus, the formation of hard tissue (1, 5). It also inhibits the inflammatory reaction resulting from the death of bacteria and promotes tooth repair (1).

Unfortunately, the existing temporary endodontic medications such as calcium hydroxide also presents some clinical disadvantages. They are permeable to tissue fluids and soluble within the root canal. Moreover, their removal from the root canals can prove to be quite difficult (6, 7). This can have consequences, as it may affect the sealing capacity of the restoration, causing leakages at the apical region of the root (8). Unremoved calcium hydroxide residues may interfere with the ability of the definitive sealer to properly penetrate all dentinal tubules (9). It can also create a physical barrier between the dentin and the endodontic sealer, thus altering its adhesion (10). This altered adhesion can also be resulting from the degradation of the collagen matrix composing the dentin, caused by the high alkalinity of the calcium hydroxide (11). In fact, the high alkalinity of calcium hydroxide is an advantage against bacteria, but it also jeopardizes the bond between the hydroxyapatite and collagen fibers, which are responsible for dentin strength. This issue can lead to a drastic decrease of the mechanical properties of the dentin structure and makes it less resistant to compressive forces, even with short-term exposure (12). Moreover, in some cases, the treatment requires multiple appointments, and the treated teeth are susceptible to fracture, coronal microleakage and esthetic demands during the long-term treatment (13).

In the 1970s, researchers started investigating bioceramics and their applications in medicine and dentistry. Bioceramics are considered as ceramic materials, along with alumina and zirconia, hydroxyapatite, bioglasses, resorbable calcium phosphates, and calcium silicate. In dentistry, bioceramics are used as sealers and cements during endodontic treatments (14–18). Their wide application is due to multiple clinical advantages. Bioceramics are biocompatible, non-toxic, chemically stable in a biological environment and easy to use (19–22). They may induce the deposition of apatite-like crystals and establish a chemical bond between living tissues and filling materials; this is particularly true when using bioactive glasses (23). They also present a good antibacterial activity due to their high pH (14, 24, 25). The physicochemical, biological, and mechanical properties of most bioceramics developed for endodontic application are considered as the most preferred properties for an ideal endodontic treatment (26).

Endodontic materials can be premixed by the supplier or provided separately in two components, which must be mixed before application; this type of material is known as paste-paste system. The use of premixed endodontic materials reduces the potential risk of heterogeneity. Indeed, if the phases are not correctly mixed, there is a risk of altering the properties of the material. Another advantage of those premixed materials is that they are easy and quick to use (14, 27).

Calcicur (Voco, Cuxhaven, Germany) is a water-based calcium dihydroxide paste. According to the manufacturer, its pH is 12.5. Studies have shown that the pH value of this material does not change significantly during the first 5 days after application (28, 29). Several studies investigated the antibacterial activity of Calcicur. It was demonstrated to have an important antibacterial effect against *Streptococcus salivarius*, *Streptococcus sanguis*, *Streptococcus mutans* (30, 31) and *Enterococcus faecalis* (32). Regarding cytocompatibility, Calcicur has a good cytocompatibility (30, 33). A novel temporary material based on calcium silicate strategy was recently introduced in the dental market. Bio-C Temp (Angelus, Brazil) was presented as a temporary intracanal medication thanks to its calcium ions releasing, pH alkaline, biocompatibility, ease of removal and high radiopacity (34). The physicochemical, antibacterial activity against *Enterococcus Faecalis*, and cytotoxicity of this premixed calcium silicate endodontic temporary medication have not been thoroughly studied and compared to the gold-standard premixed calcium hydroxide.

Therefore, the aim of the present *in vitro* study was to compare the physicochemical and biological properties of a conventional premixed calcium hydroxide cement (Calcicur) as control group to a novel temporary medication cement based on bioceramic strategy. The null hypothesis was that there would be no difference between the two tested materials in terms of physicochemical and biological properties.

## 2 Materials and methods

Calcicur “Cal” (Voco, Cuxhaven, Germany) and Bio-C® Temp “Bio-C” (Angelus, Brazil) were prepared as per manufacturer's instructions. Their composition is described in Table 1.

**Table 1:** Composition of tested materials.

Materials	Composition	Mixing
Calcicur (Cal)	Water, calcium hydroxide	Premixed
Bio-C® Temp (Bio-C)	Tricalcium silicate, dicalcium silicate, calcium oxide, base resin, calcium tungstate, polyethylene glycol, titanium oxide	Premixed

### 2.1 Specimen preparation

The preparation of the specimens was carried out using molds of different dimensions, depending on the tests performed in this study. For the evaluation of the pH, antimicrobial activity, cytotoxicity, and crystallography, Teflon molds (height: 3.8 mm; diameter: 3 mm) were used to create cylindrical specimens. For the roughness study, Teflon molds (height: 2 mm; diameter: 10 mm) were chosen. Finally, for the evaluation of solubility and wettability, stainless steel ring molds (height: 1.5 mm; diameter: 20 mm) were used.

The different materials were injected directly using their injection tip into the different molds with glass slides underneath to provide a flat surface. To allow the materials to set properly, all specimens were stored at 37°C and in a humid environment for 48 h.

### 2.2 PH measurement

Three specimens were immersed into10 ml of distilled water at 37°C. The pH of each medium was measured at 3, 24, 72 and 168 h using a pH-meter (Eutech Instrument, CyberScan pH 510, Singapore). The specimens were left at room temperature for 15 min before measurement. To perform the calibration of pH-meter, standard solutions at pH 10, 4 and 7 were used (Hanna Instruments, Lingolsheim, France). The pH-electrode was rinsed between each measurement with distilled water to eliminate the remaining previous solution.

### 2.3 Antimicrobial activity

To study the antibacterial activity of both materials, a direct contact test (DCT) was outperformed using *E.faecalis*, ATCC 29212. This was cultured in Brain Heart Infusion medium (BHI) (Darmstadt, Germany) and its turbidity adapted to DO_600nm_ = 0.3. In a culture plate, three specimens of each material were placed at the bottom of the wells and 1 ml of bacterial medium was added. A positive control was made by filling a well with the bacterial medium only. The plate was incubated for 24 h in anaerobic conditions at 37°C under constant stirring at 450 rpm. At the end of the incubation, each well was serially diluted 10-fold up to 10^6^ in BHI. Onto a BHI agar plate, 100 µl of each dilution was homogeneously spread and incubated for 24 h at 37°C. The concentration of *E.faecalis* was measured by manual counting onto the plates and their CFU/ml (colony forming units/ml) was determined.

### 2.4 Water sorption test and roughness

For the water sorption test, 4 µl drop of distilled water was placed on the surface of each specimen. A contact angle device (Bioloin Scentific Oy, Tietäjäntie, Espoo, Finland) was used to evaluate the sorption time of the drop. Three measurements for each group were analyzed after 10 s of drop contact by using a horizontal camera to track its profile.

To analyze the roughness of each material, six measurements were performed by using a 3D non-contact digital profilometer (Keyence, Osaka, Japan) at 3000× magnification. A software (Keyence 7000 VHX, Osaka, Japan) was used to calculate the roughness of each surface.

### 2.5 Morphological characterization

Six specimens of each tested material were immersed in 1× Phosphate-Buffered Saline (prepared from PBS 10x, Dominique Dutscher, Bernolsheim, France) during 24 h, 72 h or 168 h, at 37°C. After incubation, two of them were analyzed at each period. They were rinsed and dried in a fume hood for 24 h. Then, by using a Hummer JR sputtering device (Technics, California, USA), the samples were sputter-coated with gold-palladium (20/80) and examined through a scanning electron microscope (SEM) (Quanta 250 FEG scanning electron microscope; “FEI Company, Eindhoven, The Netherlands”, 10 kV) with a magnification of ×500 to investigate the crystalline formation.

### 2.6 Solubility and flow test

For solubility evaluation, the procedure described in ISO 6876:2012 was adopted. Three specimens for each material were used. The molds were placed on a glass plate and the materials were plugged into the molds. After the setting time (48 h), molds were weighed (accuracy ± 0.0001 g) three times and underwent an aging period by being immersed for 24 h in 50 ml of distilled water at 37°C. After the immersion period, the specimens were slightly rinsed with distilled water and dried for 6 h at 110°C. Each specimen was reweighed to obtain a final weight. The solubility percentage was obtained as per the difference between the initial weight of the specimens (before immersion) and their final weight.

To perform the flow test, the procedure described in ISO 6876:2012 was also used. Fifty microliter of each material was placed on a glass plate. After 3 min, the material was covered with a second glass plate and a weight of 100 g was placed on it. After 10 min, the diameter of the compressed material was measured twice. The test was performed in triplicate and the diameter values were averaged.

### 2.6 Cytotoxicity

Human periodontal ligament stem cells (PDL) were isolated from human third molars from one donor after approval. Teeth were harvested from patient attending a private oral surgery practice based in Strasbourg, and the protocol was approved by the French Ministry of higher education and research. The cells were cultured at 37°C in Alpha-MEM (Lonza) with 10% of FBS (Gibco) and 1% of penicillin−streptomycin (Biowest), further referred to as “cell culture medium”. The cytotoxicity tests were performed according to ISO 10993-5. The tested materials were incubated for 24 h with 1 ml of cell culture medium at 37°C, then the resulting extracts were used undiluted (100%) or diluted at 1/2 (50%) or 1/10 (10%) with cell culture medium. The extracts were put in contact with the cells for 24 h at 37°C, and CellTiter-Glo® Luminescent Cell Viability Assay (Promega) was used according to manufacturer's instructions. Chlorhexidine was used as a negative control treatment.

### 2.7 Statistical analysis

The data obtained was analyzed by Sigma Plot (release 11.0, Systat Software, Inc., San Jose, CA, USA). To verify the normality of the data, the Shapiro-Wilk test was applied. The Kruskal-Wallis test (one-way analysis of variance on ranks) was used when normality was not verified. Statistical significance level was set at *α* = 0.05.

## 3 Results

### 3.1 PH analysis

**Figure 1:**
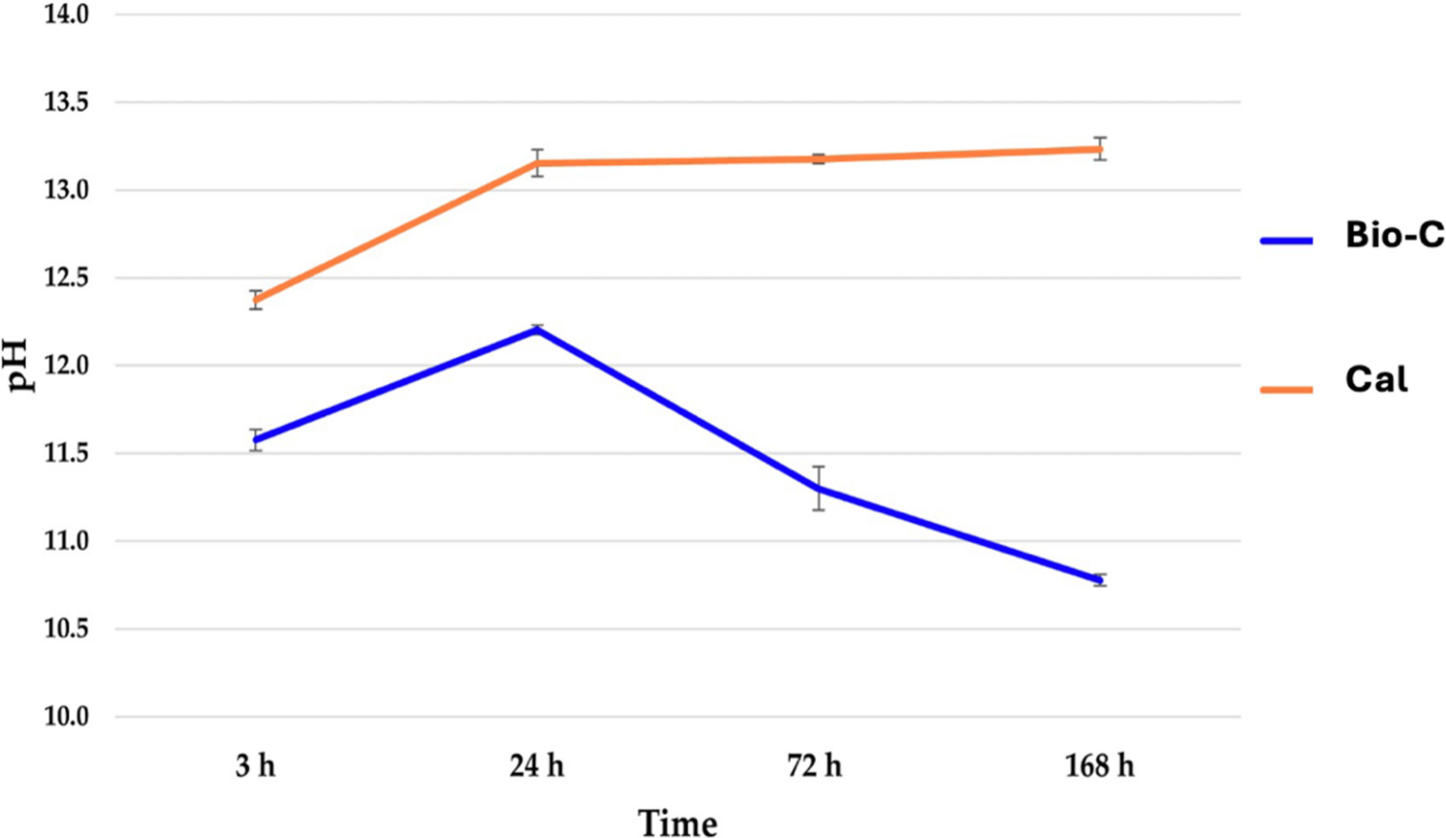
PH evolution of the distilled water after 3, 24, 72, and 168 h in contact with the tested materials, at 37°C. Bio-C® Temp (Bio-C) and Calcicur (Cal).

The pH of the storage media of the tested products over 168 h, is presented in Figure 1. Both the tested materials presented an important alkalinization activity. Cal showed a higher pH than Bio-C at 3, 72 and 168 h (*p *< 0.05). However, no statistically significant difference was found between Cal and Bio-C at 24 h (*p *> 0.05). A slightly decrease in pH for Bio-C was found after 72 h.

### 3.2 Antimicrobial activity

**Figure 2:**
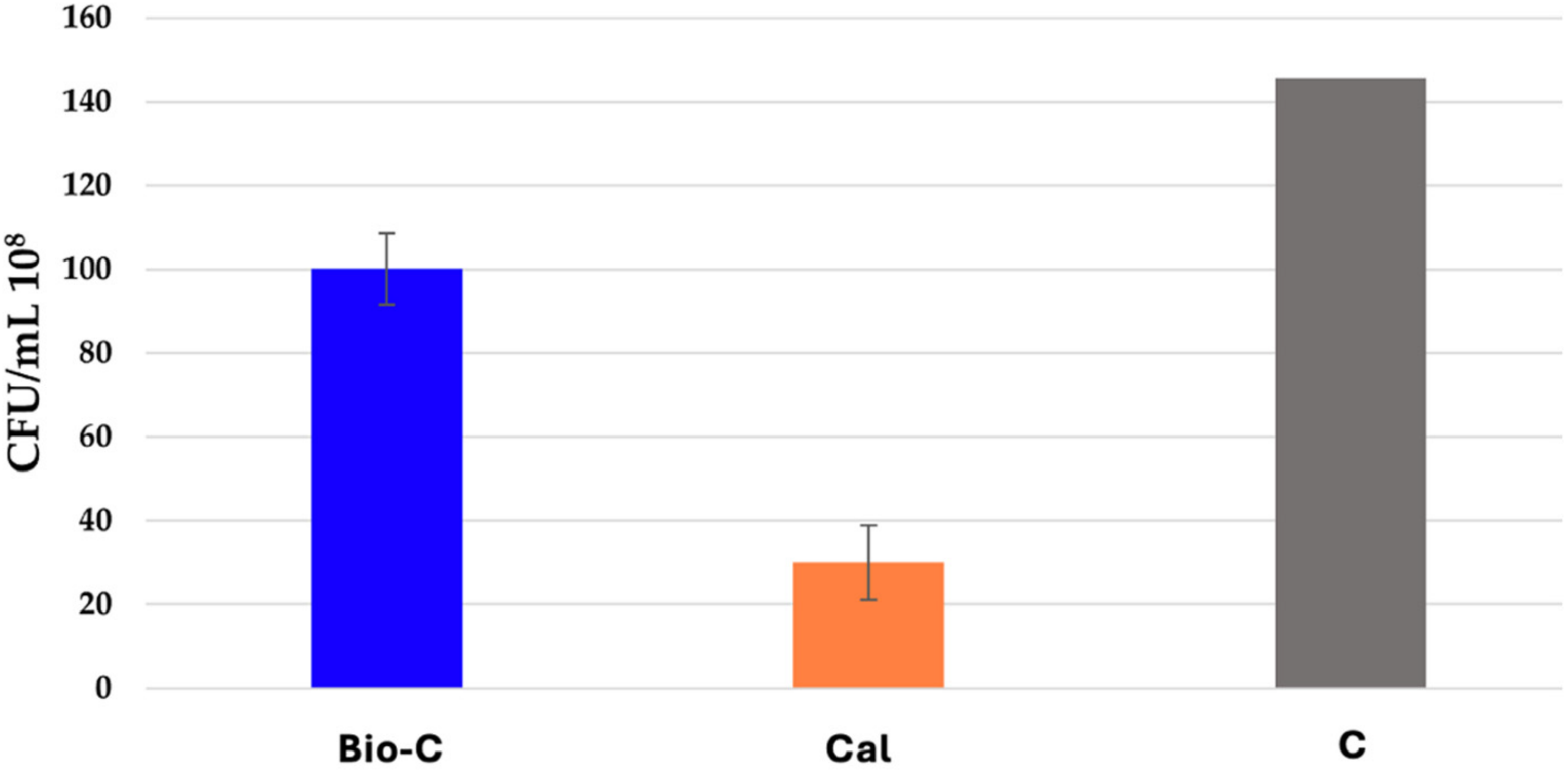
Number of colonies forming units/ml (CFU/ml) of *E. faecalis* in contact with Bio-C® Temp (Bio-C) and Calcicur (Cal) samples and the control group (Ctrl) at 37°C, after 24 h in anaerobic conditions.

Both materials had a significant antibacterial effect compared to the control group (*p* < 0.05) (Figure 2). Cal showed significant higher antibacterial activity on *E. faecalis* compared to Bio-C (*p* < 0.05), as it was able to kill 79% of bacteria. Bio-C killed only 30% of the bacteria.

### 3.3 Water sorption test and roughness

**Figure 3:**
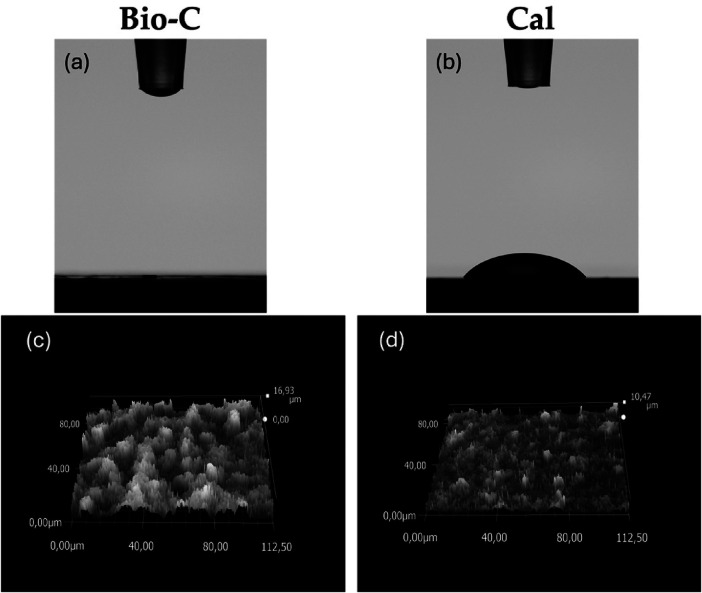
**(A,B)** contact angles between a 4 µl drop of distilled water and the surface of the tested samples, 10 s after deposition. **(C,D)** Roughness of each sample demonstrated by digital micrographs of samples surface by Keyence 7000 VHX. Bio-C® Temp (Bio-C) and Calcicur® (Cal).

At 10 s, significant higher contact angles were observed for Cal (64.6 ± 4.53°) compared to Bio-C (0°) (*p *< 0.05) (Table 2, Figure 3).

**Table 2:** Contact angles of a 4 µl drop of distilled water onto the material surfaces, after 10 s of deposition.

Test\Materials	Bio-C	Cal	*p* < 0.05
Contact angle (°)	0 ± 0^a^	64.6 ± 4.53^b^	a < b
Roughness (Sa)	2.39 ± 0.5	1.80 ± 0.75	No

Roughness means and standard deviations (Sa) of the tested materials. Bio-C® Temp (Bio-C) and Calcicur (Cal). Superscript letters (^a, b^) indicate statistical significances (*p *< 0.05).

The surface roughness of the specimens is presented in Table 2 and Figure 3. No statistically significant difference was found between Bio-C and Cal samples (*p* > 0.05).

### 3.4 Morphological characteristics

**Figure 4:**
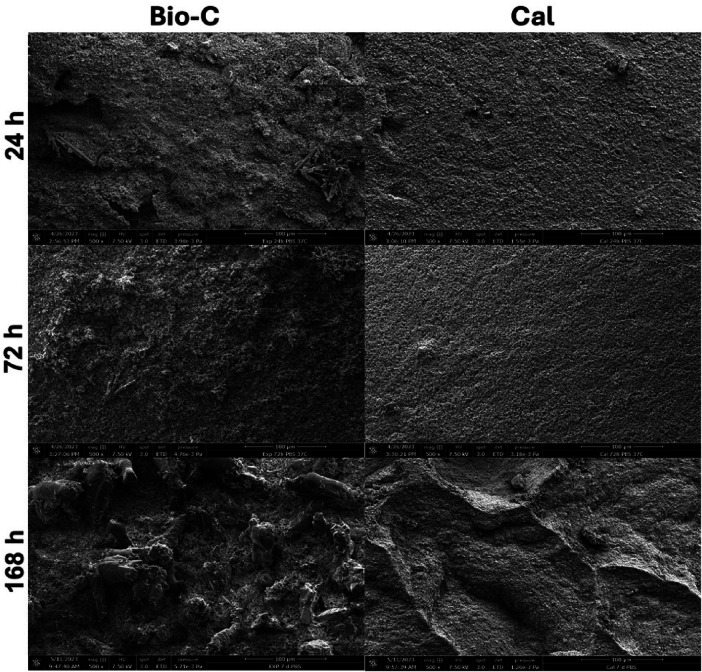
Scanning electron microscope images (SEM) (500× magnification) showed the morphological evolution of each material surface after 24, 72 and 168 h in phosphate-buffer solution at 37°C. Bio-C® Temp (Bio-C) and Calcicur (Cal).

**Figure 5:**
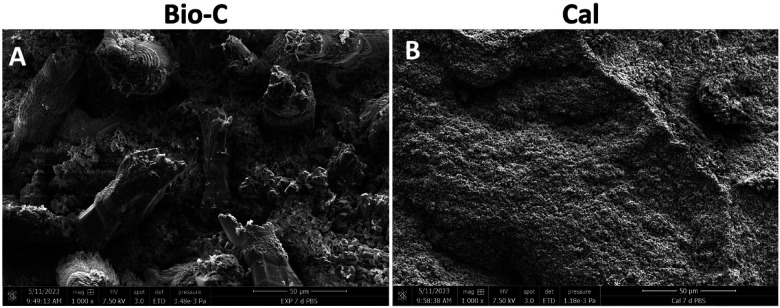
Scanning electron microscope images (SEM) (**A** and **B**: 1000× magnification) showed the morphological evolution of each material surface after 168 h in phosphate-buffer solution at 37°C. Bio-C® Temp (Bio-C) and Calcicur (Cal).

The crystalline structures of the specimens are shown in Figure 4. After 24 and 72 h, Bio-C showed the formation of small and elongated crystalline structures. After 168 h, these structures were larger and more numerous. Concerning Cal, small crystalline structure formation onto the surface was observed at 7 days (Figure 4). However, at 168 h (Figure 5), Larger crystalline structures were detected onto Bio-C surface while micro crystalline structures were detected onto Cal surface after 7 days of incubation in PBS at 37°C.

### 3.5 Solubility and flow test

The solubility means and standard deviations of both tested materials are presented in Table 3. Higher significant difference was found for Bio-C compared to Cal (*p *< 0.05). Flow test results are given in Table 3. Higher statistically flowability was found for Bio-C compared to Cal (*p* < 0.05).

**Table 3:** Solubility means and standard deviations (*n* = 3) in distilled water at 37°C and flow test (*n* = 3) of Bio-C® temp (Bio-C) and calcicur (Cal).

Solubility (%)		*p* < 0.05
Bio-C	Cal	
12.25 ± 0.38^a^	6.89 ± 2.22^b^	a > b
Flow (mm)		
Bio-C	Cal	
362 ± 17^c^	138 ± 27^d^	c > d

Superscript letters (^a–d^) indicate statistical significances (*p *< 0.05).

### 3.6 Cytotoxicity

**Figure 6:**
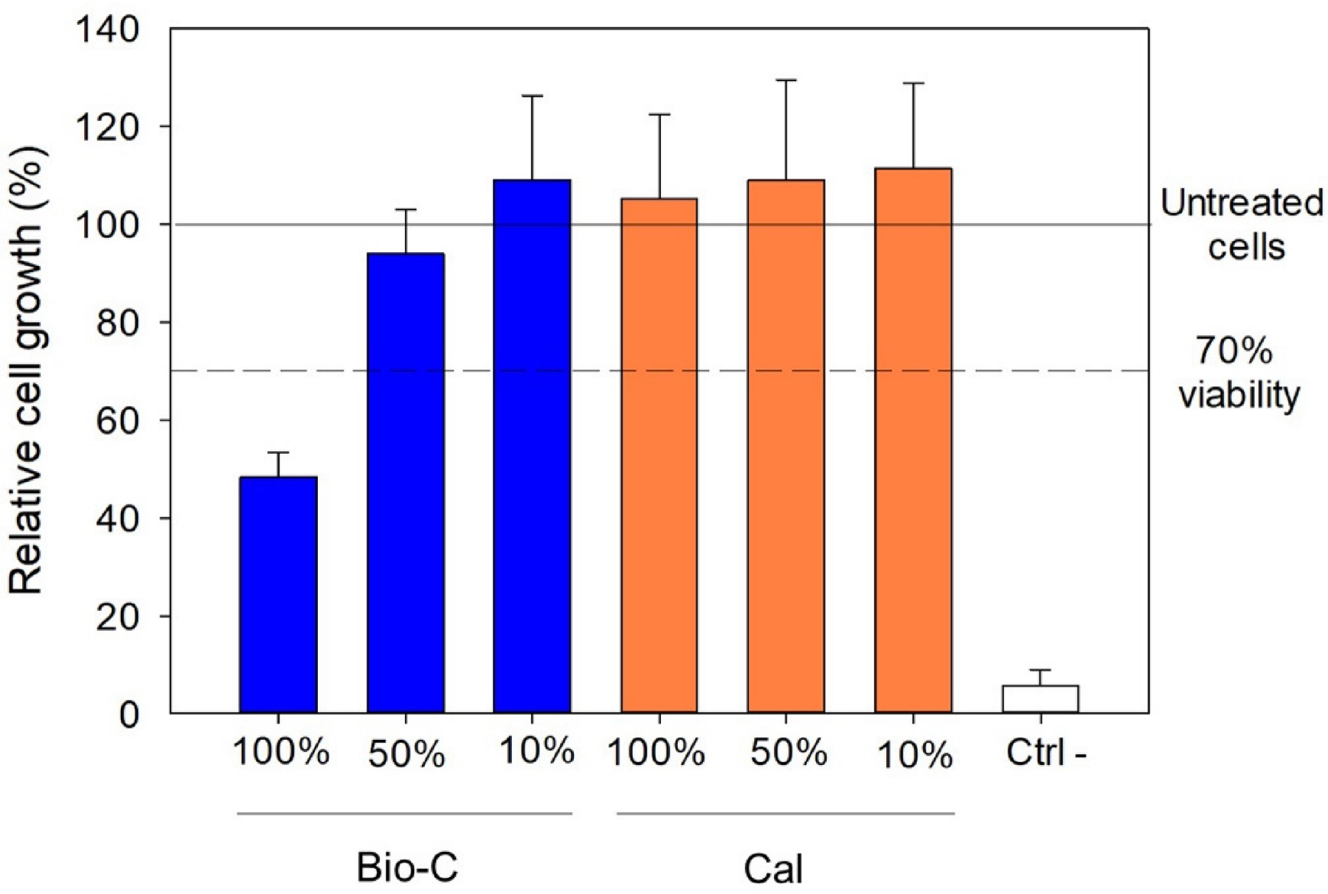
*In vitro* cytotoxicity test. Cell growth was evaluated using CellTiter-Glo® luminescent cell viability assay. The results are expressed as a percentage, where 100% correspond to untreated cells. The results represent averages from three independent experiments +/− SD. Bio-C® Temp (Bio-C), Calcicur (Cal) and negative control group (Ctrl-).

The results of cytotoxicity testing demonstrate that Bio-C at 100% was slightly cytotoxic with viability decrease to 48.37% (cytotoxicity threshold: 70%). All the other conditions were found non-cytotoxic at any concentration (viability >70%). Thus, Bio-C shows more cytotoxicity compared to Cal, when concentrated (Figure 6).

## 4 Discussion

High alkalinity is an essential element for a material that is used as a temporary medication in endodontic treatment. A high and stable pH is responsible for antibacterial activity (5), activation of repair and remineralization, neutralizing lactic acid, thus avoiding mineral decomposition of dentin components and inducing hard tissue formation (1). In this study, both materials showed an alkaline pH (Figure 1), but the traditional Calcium hydroxide cement showed a significant higher and a more stable pH for 7 days when compared to Bio-C. Therefore, this outcome is enough to reject the null hypothesis of this study. Kharouf et al. (18) showed a decrease in pH of the storage solution of the bioceramic materials and they declared that such a decrease could be related to the fact that OH^−^ in the solution could be reabsorbed at the surface of the cement to produce mineral deposition. Moreover, Zhang, H et al. (35) showed that *bacteria* are eliminated in the first 2 min of exposure to a bioceramic materials, due to its high alkalinity.

In addition, the pH and the released Ca ^2+^ directly influences the antibacterial activity of the material (36). The hydroxyl ion, released after the dissolution of calcium hydroxide, is the main cause of this activity (1). Bio-C contains calcium oxide and is therefore able to form calcium hydroxide when in contact with water (37). Hydroxyl ions are also released from Bio-C. This is a powerful oxidative radical that can cause damage to DNA of bacteria and to its cytoplasmic membrane, as well as protein denaturation. All these effects alter the vital functions of the bacteria (cell division and growth, wall formation, lipids biosynthesis, etc.) and lead to the death of the bacteria (1). In the present work, *E. faecalis* was used because it is a facultative Gram-positive anaerobic bacterium, frequently found in necrotic dental pulp (38, 39); it is also highly resistant to harsh conditions (stress, heat, UV, sodium hypochlorite (40). The results of DCT carried out in this study (Figure 2) are coherent with those obtained from pH variations, as the material with the highest antibacterial activity was Cal, which had a more alkaline and constant pH. The results of the current study are in accordance with previous works that demonstrated the effectiveness of Cal against *E.faecalis* (32). In the few other studies about Bio-C, it was also reported that a bioceramic based-material had less antibacterial activity than Calcium-hydroxide based intracanal materials. The authors suggested that this difference could be attributed to the different vehicles composing the pastes, and to fewer CH molecules formed in the hydration reaction (41–43). It should be noted that in the study of Guerreiro JC, the crystal violet test was used to evaluate the action of different materials on biofilm biomass, which could be more relevant to the clinical situation. The fact that crystal violet stains living cells, dead cells, and the biofilm matrix equally is a drawback of this test. As a result, it is unable to distinguish between living and dead cells (41).

The solubility of the tested materials could be related directly to their bioactivity, and it can influence the pH (28, 44), the release of calcium and hydroxyl ions over time, and the sealing of the definitive obturation (8). The solubility of the materials was studied according to the ISO 6876:2012 standard. In this work, higher solubility was found for Bio-C compared to Cal (*p *< 0.05). In contrast to the pH values, where Cal demonstrated higher pH compared to Bio-C, the solubility of Cal was less evident than Bio-C (*p* < 0.05). This could be due to the fact that several dissolved elements from Bio-C had no influence on the pH and the antibacterial activity (21). In addition, despite the lower solubility of Cal, it showed higher pH, so we can hypothesize that higher Ca^2+^ ion and OH^−^ were released from Cal compared to Bio-C.

The surface analysis performed by SEM in both tested materials after immersion in PBS revealed different crystallographic profiles (Figure 4). Elongated crystalline structures were observed on the surface of Bio-C after 7 days of immersion in PBS. In order to know the chemical composition of the crystals, an EDX analysis should have been conducted; this represents a limitation of this *in vitro* study. Micro and nano crystalline structures were observed on the surface of Cal, after 7 days in PBS. Accordingly, Stefanova et al. (45) showed in their study the same morphological characteristics for a calcium hydroxide material. Several studies have also shown that calcium silicates can induce the formation of crystals on their surfaces (46–48). Therefore, the mineral deposition observed on Bio-C may have an important role in the remineralization and healing process when it is used as an endodontic material.

Greater flowability was detected for Bio-C compared to Cal (*p* < 0.05) (Table 3). This characteristic may play an important role in the obturation quality and the ability of penetration of endodontic materials into dentinal tubules, which is recommended to entomb bacteria and kill the remaining microorganisms in the different anatomical parts of the root canal system (36, 49). Therefore, it is speculated that Bio-C could fill the canal and obturate the anatomical complexity easier than Cal. Further studies should be performed to evaluate the quality of obturation of the novel product and the ability of removing it from the root canal in case of endodontics retreatments.

During the contact angle test performed on Bio-C and on Cal, it was observed that the wettability of Bio-C was significantly higher than Cal (Table 2, Figure 3). The higher hydrophilicity of the endodontic material may play an important role in their cell attachment and biocompatibility (21, 22). The contact angle can change depending on the roughness and chemical composition of the surface (50, 51). The surface roughness of the specimens was therefore evaluated (Table 2, Figure 3). The roughness of Bio-C and Cal was similar, and no significant difference was encountered (*p* > 0.05). The rapid adsorption of the drop on the surface of Bio-C may reflect a good biocompatibility; it may be correlated to its chemical composition and the porosity which should be investigated in further research.

Regarding the cytotoxicity results obtained in this study, at 10% and 50% of extract dilution, no statistically significant difference was found between both products (*p* > 0.05). In contrast, at 100% (undiluted extract), Bio-C showed a slight cytotoxicity with viability decrease to 48.37% (cytotoxicity threshold: 70%). This could be attributed to the higher solubility of Bio-C and the different released elements, which could be toxic to this type of cells such resin base that is included in the chemical composition of Bio-C. Moreover, the cell responses could be related with various factors including the cell line, the material state, the experimental period, etc. (52). The cytotoxicity of Bio-C was also found worrying in the studies of Villa et al. and Capitanio et al. (42, 43), whereas in the study of Guerreiro et al. (41), Bio-C had similar cytocompatibility at higher dilution, when compared with two calcium hydroxide intracanl medicament, Calen and UltraCal XS.

The findings of this study are encouraging for the application of an intracanal medication based on bioceramic strategy and calcium hydroxide in multiple visit endodontic treatment. Clinically, the use of these product based on calcium silicate and calcium hydroxide play an important role in killing the bacteria in root canals especially between appointments (53). Moreover, the use of intracanal medicaments minimizes ingress of pathogens through a leaking restoration and could be useful in complex cases with pulpal necrosis and apical periodontitis (53). Bio-C has acceptable biological activities except for its concentration at 100% which was slightly cytotoxic with viability decrease to 48.37% (cytotoxicity threshold: 70%).

Regarding the removal of Bio-C from the root canal, it was suggested to use a combination of sodium hypochlorite and Passive ultrasonic irrigation (43). In our study, we did not investigate the residues of Bio-C on dentine substrate, and this should be further investigated. According to the findings of previous studies, it was even stipulated that Residues of bio-C improved the adaptation of a calcium silicate-based sealer (43). These findings should be verified in future studies.

One of the limitations of the present study is the sample size for each test. Moreover, longer contact time of each material with PBS should be performed as well as with contact with other solutions. In addition, the effect of these materials on dentin structure should be analyzed. Further studies are needed for Bio-C to facilitate its complete elimination from root canal before the permanent obturation without negative impacts on the dentin.

## 5 Conclusions

Within the limitations of this *in vitro* study, physicochemical and biological properties evaluations showed that both products had an alkaline pH and antibacterial activity. Bio-C showed physicochemical properties comparable to those of the traditional endodontic medication with higher cytotoxic effect at 100% of concentration compared to the traditional one. These findings permit to consider this product as an alternative to calcium hydroxide material in endodontic medication. Further studies on the removal ability of the novel bioceramic should be performed.

## Data Availability

The raw data supporting the conclusions of this article will be made available by the authors, without undue reservation.
